# Interleukin-2 Receptor as a Marker of Oxidative Stress in Paediatric Patients with Chronic Kidney Disease or Hypertension

**DOI:** 10.3390/children12050569

**Published:** 2025-04-28

**Authors:** Nataša Marčun Varda, Mirjam Močnik, Martina Filipič, Evgenija Homšak, Mateja Svetej, Sonja Golob Jančič

**Affiliations:** 1 Department of Paediatrics, University Medical Centre Maribor, Ljubljanska ulica 5, 2000 Maribor, Slovenia; mirjam.mocnik@ukc-mb.si (M.M.); martina.filipic@ukc-mb.si (M.F.); sonja.golobjancic@ukc-mb.si (S.G.J.); 2 Medical Faculty, University of Maribor, Taborska 8, 2000 Maribor, Slovenia; 3 Department of Laboratory Diagnostics, University Medical Centre Maribor, Ljubljanska ulica 5, 2000 Maribor, Slovenia; evgenija.homsak@ukc-mb.si (E.H.); mateja.svetej@ukc-mb.si (M.S.)

**Keywords:** inflammation, cardiovascular risk, children, interleukin-2 receptor

## Abstract

**Background/Objectives**: Oxidative stress and systemic inflammation are significant contributors to the development and progression of cardiovascular disease, causing adverse effects on vascular health and atherosclerosis from an early age. Patients with established cardiovascular risk factors commonly exhibit markers indicating heightened oxidative stress and inflammation. Our study sought to assess the levels of interleukin-2 receptor, which could serve as an early indicator of cardiovascular damage due to oxidative stress and inflammation in at-risk children. **Methods**: The study comprised 46 paediatric patients with chronic kidney disease, 50 paediatric patients with hypertension, and 33 healthy controls. Anthropometric measurements, pulse wave velocity, body composition, routine laboratory tests, and measurements of interleukin-2 receptor levels were conducted for all participants. **Results**: Interleukin-2 receptor levels were notably lower in patients with hypertension (*p* < 0.001) and those with overweight/obesity (*p* < 0.001) with several associated measures. Interleukin-2 receptor levels exhibited significant negative correlations with various anthropometric measurements, body composition, and liver damage and a positive correlation with kidney function tests. **Conclusions**: Children diagnosed with hypertension or obesity exhibited notably lower interleukin-2 receptor levels.

## 1. Introduction

Oxidative stress is characterised by a disruption in the equilibrium between free radicals and antioxidant defences. This imbalance, marked by an abundance of free radicals resulting from either heightened generation or insufficient antioxidant activity, prompts their interaction with proteins, lipids, and genetic material in the nucleus or mitochondria, leading to cellular harm. Studies indicate a strong correlation between free radicals and endothelial dysfunction, contributing to the development of atherosclerosis [1,2]. Understanding the roles of cytokines as messengers of inflammation has revealed a mechanism by which risk factors for atherosclerosis can influence arterial biology. The revelation that vascular wall cells are capable of producing cytokines, which are protein mediators of inflammation and immunity, offered a significant insight into the onset of atherosclerosis [3,4]. The discovery of interleukin-2 (IL-2) made it possible to generate, culture, and study T lymphocytes, where the inflammatory consequences of IL-2 are most prominent [5]. T lymphocytes were present in significant numbers in the atherosclerotic plaque, making both IL-2 and its receptor (IL-2R), among other interleukins, a potential marker of accelerated atherosclerosis [6].

While clinical symptoms of cardiovascular diseases like myocardial infarction, stroke, and peripheral vascular disease typically manifest in middle age, the onset of atherosclerosis can occur as early as in childhood. The development and progression of atherosclerosis during youth are influenced by various risk factors. Current evidence suggests that primary prevention efforts targeting atherosclerotic disease should commence in childhood. Identifying children at risk for atherosclerosis enables early intervention strategies aimed at mitigating its progression, thereby potentially preventing or postponing the onset of cardiovascular manifestations [7]. Elevated levels of inflammation have been validated in paediatric patients presenting with different cardiovascular risk factors, including chronic kidney disease [8,9]. Additionally, oxidative stress has been linked to dyslipidaemia and hypertension in these patients [8,10]. Low-level inflammation contributes to the development of primary hypertension and damage to target organs, particularly highlighting the vascular inflammatory processes observed in children before systemic inflammatory alterations occur [11]. Obesity-induced oxidative stress stands out as one of the most clearly demonstrated among cardiovascular risk factors. Adipose tissue, being a metabolically active organ, generates various inflammatory cytokines and acute-phase reactants that are linked to obesity-related comorbidities such as insulin resistance, type 2 diabetes, atherosclerotic heart disease, non-alcoholic fatty liver disease, hypertension, and hyperlipidaemia. These factors further contribute to the promotion of oxidative stress and the progression of atherosclerosis [12,13,14,15].

Therefore, the search for an oxidative stress marker should commence in childhood, aiming to recognise at-risk children with increased systemic inflammation and atherosclerosis. The aim of our study was to determine levels of interleukin-2 receptor (IL-2Rα) in children with increased cardiovascular risk, namely, with either chronic kidney disease (CKD) or hypertension (HTN), compared to healthy controls and to correlate them to several anthropometric and laboratory measures.

## 2. Materials and Methods

This research builds upon a previously published study investigating liver and kidney elastography within the same group of children [16]. It involved the participation of 46 paediatric patients with CKD, 50 paediatric patients with primary HTN (secondary causes were excluded), and 33 healthy individuals serving as controls. CKD was characterised by the presence of structural or functional kidney abnormalities persisting for more than three months. Diagnosis of HTN adhered to established diagnostic criteria and age-specific threshold values [17]. The selection process for healthy controls involved the exclusion of individuals with acute or chronic illnesses, with specific attention given to evaluating the presence of cardiovascular risk factors, obesity, and infectious or inflammatory conditions.

Parents, legal guardians, or adult participants were given written information about the research protocol and subsequently provided informed consent by signing a declaration to participate. The study followed the principles outlined in the Declaration of Helsinki and obtained approval from both the Medical Ethics Commission of the University Medical Centre Maribor (UKC-MB-KME-35/20) and the Medical Ethics Commission of the Republic of Slovenia (0210-372/2020/6).

All participants followed the same procedure: initial data entry was followed by anthropometric measurements (height, weight, waist, and hip circumference) and subsequent calculation of body mass index (BMI). Furthermore, participants were categorised into overweight/obese (BMI exceeding the 85th percentile for sex and age) or normal-weight groups. Next, body composition was assessed using bioimpedance methodology (Nutrilab Bioimpedance, Pisa, Italy, Akern 2016 [18]). In accordance with the manufacturer’s guidelines, the measurement was conducted under fasting conditions, with an empty bladder, and in a consistent lying position, steady environment, with electrodes placed identically. The analysis provided individual results for each individual’s body composition, including phase angle (PA), fat mass (FM), fat-free mass (FFM), body cell mass (BCM), total body water (TBW), and extracellular water (ECW). Blood pressure was measured utilising the oscillometric technique (Omron Healthcare Co., Kyoto, Japan). In all participants, pulse wave velocity was measured using applanation tonometry (SphygmoCor Cardiovascular Management Suite^®^, Sidney, Australia). Finally, blood samples were collected for fundamental laboratory analyses related to liver damage, kidney function, and lipid profile, alongside the determination of serum IL-2Rv using the enzyme-linked immunosorbent assay (ELISA) method.

All participants had undergone liver and kidney elastography as part of another study, and we additionally examined these results in association with the laboratory measurements of IL-2Rα.

The statistical analysis was conducted using the SPSS Statistics program (IBM, version 22). Due to the non-normal distribution of variables, as indicated by the Kolmogorov–Smirnov and Shapiro–Wilk tests, results are depicted using the median and interquartile range (IQR). Consequently, non-parametric tests were employed, including the Mann–Whitney and Kruskal–Wallis tests for group comparisons and the Spearman correlation coefficient to investigate associations between the studied markers and other variables. A multivariate regression analysis was performed to adjust for potential confounding. A significance level of *p* < 0.05 was considered statistically significant. A post hoc power analysis was performed using G*Power software, version 3.1 (Erdfelder, Faul & Buchner, Weinheim, Germany).

## 3. Results

Several fundamental findings have been previously published [16,19], which, in summary, demonstrate that groups were balanced in terms of age distribution but not in terms of sex distribution. In both research groups, boys were more prevalent, whereas in the control group, there were more girls. BMI was increased significantly in patients with HTN, and moderately in patients with CKD, where obesity was not an exclusion factor as in the control group. Among 50 patients with HTN, 36 were overweight/obese, indicating a high proportion of obesity-related HTN. This was further supported by body composition measurements, which revealed a notable increase in fat mass among patients with HTN. Due to prominent differences in anthropometric measurements and excessive weight in patients, all subjects were again divided into two groups—51 patients were overweight/obese and 78 of normal weight. The severity of CKD, along with the underlying diagnosis for each patient, has been detailed in the previous publication [16]. Most children had CKD stage 1 or 2, as the aim of the study was to identify markers of cardiovascular/kidney damage early in CKD, where complications (anaemia, underweight, metabolic-bone disease, hypertension) are not usually present.

Basic laboratory results were also collected, showing elevated kidney function markers (creatinine, cystatin C, urinary albumin/creatinine) in patients with CKD and increased indicators of liver damage (aspartate aminotransferase (AST), alanine transaminase (ALT), gamma-glutamyl transferase (GGT)) and lipid profile among patients with HTN compared to healthy controls. Liver elastography parameters were elevated in both patient groups compared to the control group, while kidney elastography parameters did not show a similar trend. However, after stratifying by weight status, liver elastography worsened, accompanied by a significant increase in kidney elastography parameters among obese participants [16,19].

In the present study, IL-2Rα was measured and was significantly lower in patients with HTN and remained lower after weight status stratification, as detailed in Table 1. A post hoc power analysis indicated that the comparison between the CKD/HTN group and the control group had insufficient statistical power (Power = 0.56). Although the power improved when participants were stratified based on obesity status (Power = 0.77), it still did not reach the threshold for adequate power.

Next, IL-2Rα levels were correlated with several researched variables, presented in Table 2. IL-2Rα unexpectedly correlated negatively with age and several anthropometric measurements, body composition parameters, systolic pressure, liver enzymes, urate, triglycerides, and liver and kidney elastography and positively with high-density cholesterol (HDL), urea, cystatin C, and urinary albumin/creatinine ratio.

**Figure 1:**
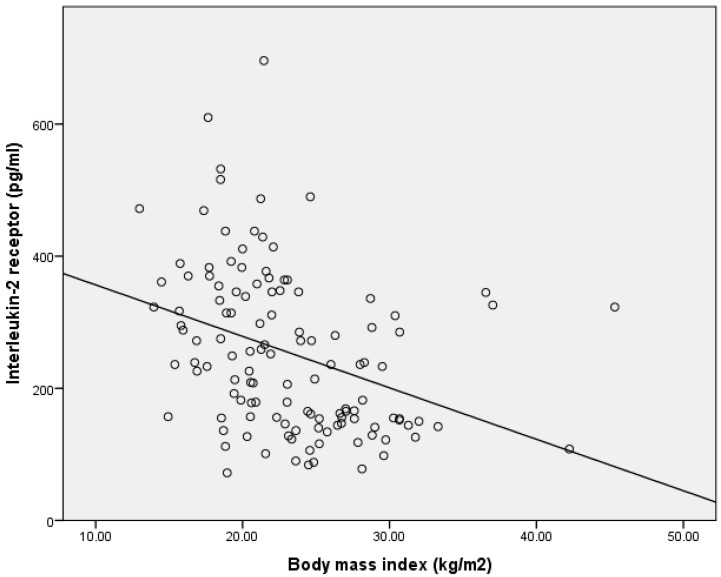
Correlation between interleukin-2 receptor and body mass index using Spearman correlation coefficient.

Excess weight seems to affect IL-2Rα levels significantly, as presented with its correlation with BMI across all participants in Figure 1. A similar trend is observed also in correlations with weight, waist and hip circumference, fat mass, and systolic pressure.

In some patients, carotid intima-media thickness (CIMT) was measured as part of routine investigations. CIMT was measured in 19/46 patients with CKD and in 48/50 patients with HTN. The correlation, using the Spearman correlation coefficient, between left and right CIMT and IL-2Rα was weak and insignificant (r = −0.022, *p* = 0.864 in right CIMT; r = 0.006, *p* = 0.965 in left CIMT).

As the high prevalence of obesity in the HTN group introduces potential confounding, a multivariate analysis was performed with IL-2Rα as the independent factor. Dependent factors included markers of hypertension (systolic and diastolic blood pressure) or CKD (creatinine, cystatin C, urinary albumin/creatinine), adjusted for body mass index. The model was statistically significant, with *p* < 0.001, and fitted moderately with R^2^ = 0.25. Beta coefficients are presented in Table 3 and show that systolic pressure is a strong negative predictor of IL-2Rα; meanwhile, cystatin C is a positive predictor. They also confirm that higher BMI is linked to lower IL-2Rα levels.

## 4. Discussion

This study aimed to evaluate IL-2Rα in paediatric patients with CKD or HTN compared to healthy controls. IL-2Rα was significantly lower in patients with HTN and remained lower after weight status stratification. Weight and BMI correlated negatively significantly with several cardiovascular risk measures—the opposite of what was expected according to the proinflammatory role of IL-2Rα.

IL-2 is a proinflammatory cytokine and induces proliferation of T cells and stimulates natural killer cells and B cells to divide and produce antibodies. By doing so, its elevated levels were associated with inflammation, characteristic for atherosclerosis. Subsequently, increased levels of IL-2 were associated with adult patients with coronary artery disease [20] and CIMT [21].

IL-2 binds to IL-2R, which is composed of three different chains: α, β, and γ, which can be expressed separately and differently on various cell types. After IL-2 binds to the receptor, its complex is internalised, β and γ chains are degraded, but α chain is recycled to the cell surface and shed to circulation [22,23]. According to the proposed mechanism, its levels were evaluated in previous studies and were associated positively with several baseline cardiovascular risk factors, subclinical cardiovascular disease, and events in adults [24]. Also, in children, studies confirmed its elevated levels in children with Kawasaki disease [25]. IL-2 receptors are expressed abundantly also in cases of malignant diseases in children, where elevated levels are likely, at least in solid tumours, to result from the normal peripheral immune response to growth of the neoplasm, or are released from activated lymphoid cells [26].

Therefore, our results for IL-2Rα are contradictory to general findings so far. The levels of IL-2Rα were significantly lowered in paediatric patients with hypertension, where obesity-related hypertension predominates. Indeed, after stratification for weight status, the difference remained, with significantly lowered levels of IL-2Rα in patients with overweight/obesity. Furthermore, several correlations indicate a trend of lowering, e.g. a negative correlation between the concentration of IL-2Rα and some investigated cardiovascular risk factors. Interestingly, some positive correlations were also detected between IL-2Rα and kidney function tests, albuminuria, and HDL. The last is in accordance with determined negative correlations due to HDL’s protective function.

Our results indicate that IL-2-mediated inflammation could play a role in kidney damage, which was demonstrated also in adult studies [27] but has not been researched extensively in the paediatric population. Children with CKD exhibit increased serum levels of proinflammatory cytokines [28]. IL-2 and IL-2Rα were increased in children on haemodialysis [29]; however, another study found no difference in IL-2 in children with chronic renal failure [30]. It is important to emphasise that our children with CKD had only mild disease; nevertheless, the correlation between IL-2Rα and kidney function was significant for urea, cystatin C, and albuminuria.

However, with the obesity presence in the paediatric population, IL-2-mediated inflammation might be even reduced. Low-grade inflammation is a hallmark of adult obesity, with adipose tissue depots producing and secreting inflammatory mediators, most frequently IL-6, interleukin-8 (IL-8), interleukin-1 receptor antagonist (IL-1Ra), and TNF-α, among others [31,32,33,34]. Some studies confirmed the proinflammatory role of IL-2 in patients with obesity [35,36]; others found no difference between the obese and lean with the decrement of physical activity [37], with one even finding a decrease in IL-2 in obese participants [38], in accordance with our study. This might be the consequence of a specific inflammatory response in obesity that has yet to be elucidated in detail. By gaining a deeper understanding of IL-2Rα dynamics, we may be able to identify paediatric patients with accelerated inflammation, making this marker potentially useful from an early age. The influence of IL-2 on the homeostasis of the immune response is emphasised with the IL-2 effect on regulatory T cells (Treg cells), leading to the contribution of low levels of IL-2 to the persistent inflammatory stage. Specifically, a low or absent concentration of IL-2 can induce decreased Treg cells and failure to control autoreactive and inflammatory responses by inducing over-activity of the T-cell response [38]. In addition, the shedding of the IL-2Rα chain might serve as a negative feedback mechanism to regulate immune activation. Soluble IL-2R, which retains a low affinity for IL-2, can bind and sequester IL-2, thereby reducing its availability and limiting the proliferation of conventional T cells. In contrast, Treg cells, which express the high-affinity IL-2 receptor, would remain largely unaffected—unless the levels of soluble IL-2R become so high that they completely deplete IL-2 from the local environment. This scenario would shift the balance toward immune tolerance rather than sustained immune activation.

Down-regulation of IL-2 in obesity has been shown also in studying human preadipocytes and adipocytes [39]. Therefore, a low level of IL-2 and its receptor IL-2Rα could represent early stages of inflammation, characteristic for the paediatric population. Similarly, lowered IL-2 levels were found in prepubertal obese children [40] and children with insulin-dependent diabetes mellitus [41]; however, there was no difference in levels in boys with increased BMI [42], or they were even elevated in another study in children with obesity [43], showing the need for further studies.

An important limitation of our study is the small participant number, which reduces the statistical power, as seen through post hoc power analysis. The cohort used was the same as in prior elastography studies, which provides potential bias, namely, excluding younger and non-cooperative patients. Also, a reliable measure of atherosclerosis is lacking to provide reference to our results. This was attempted to be achieved with pulse wave velocity and CIMT measurements, measures of subclinical atherosclerosis [44]. However, no associations were demonstrated between measured parameters, and several limitations lie in determining the pulse wave velocity in children [45], making this method less suitable as a gold standard for atherosclerosis evaluation in children. Another important aspect is also the fact that inflammation and increased oxidative stress have an effect in HTN, obesity, and CKD, making it difficult to compare the groups when some of the underlying causes of inflammation and oxidative stress overlap. Given the multifactorial nature of both processes, it is not feasible to clearly differentiate their individual contributions. Although we performed a multivariate regression analysis to adjust for excess weight, the inherently complex and overlapping mechanisms of inflammation make it difficult to isolate specific effects. Moreover, numerous unmeasured or unmeasurable factors—such as genetic predisposition or environmental pollutants—may also influence inflammatory responses. The complexity of these interactions makes a strict distinction virtually impossible. This complexity, however, is precisely what makes our findings so compelling: the observed dynamics vary according to obesity status and blood pressure (showing negative associations), in contrast to the positive associations seen with kidney function markers. These divergent patterns suggest distinct underlying mechanisms in the paediatric population, warranting further investigation.

## 5. Conclusions

In conclusion, IL-2Rα is a potential marker for cardiovascular risk and atherosclerosis-associated inflammation assessment in children. According to our results, IL-2 might be down-regulated in children with obesity with negative correlations with several adiposity measures and associated laboratory results affecting low-grade inflammation in early stages of obesity. However, positive associations with kidney function markers (urea, cystatin C, and albuminuria) imply a proinflammatory role of IL-2, as known in adults. These limited results therefore show that while serum IL-2R has been increasingly evaluated, the possible function of this molecule has not been fully elucidated. Our results might reflect the paradoxical function of the IL-2–IL2R pathway in immunity and self-tolerance, as well as the possible diverse function of IL-2.

## Figures and Tables

**Table 1:** Interleukin-2 receptor concentration between different groups. Results are presented as median (IQR) with comparison to control group using Mann–Whitney test and Kruskal–Wallis test to detect differences between all three groups in the first part of the table. CKD—chronic kidney disease, HTN—hypertension, KW—Kruskal–Wallis test, MW—Mann–Whitney test, IL-2Rα—interleukin-2 receptor.

Variable	CKD Group (N = 46) MW	HTN Group (N = 50) MW	Control Group (N = 33)	KW
IL-2Rα (pg/mL)	232 (118) *p* = 0.160	147 (35) ***p* < 0.001**	295 (172)	***p*** **< 0.001**
	**Overweight/Obesity (N = 51)**		**Normal Weight (N = 78)**	**Comparison (MW)**
IL-2Rα (pg/mL)	162 (119)		286.5 (186)	***p*** **< 0.001**

**Table 2:** Correlations between interleukin-2 receptor with other variables across all participants using Spearman correlation coefficient (r). BMI—body mass index, FFM—fat-free mass, TBW—total body water, ECW—extracellular water, BCM—body cell mass, FM—fat mass, PA—phase angle, AST—aspartate aminotransferase, ALT—alanine transaminase, GGT—gamma-glutamyl transferase, LDL—low-density lipoprotein, HDL—high-density lipoprotein.

Anthropometric Measurements, Body Composition, Blood Pressure, Elastography Measurements		Laboratory Measurements	
Variable	IL-2Rα	Variable	IL-2Rα
**Age**	**r = −0.188** ***p* = 0.038**	AST	r = 0.047 *p* = 0.606
**Height**	**r = −0.256** ***p* = 0.004**	ALT	**r = −0.178** ***p* = 0.049**
**Weight**	**r = −0.447** ***p* < 0.001**	GGT	**r = −0.265** ***p* = 0.003**
**BMI**	**r = −0.443** ***p* < 0.001**	Urea	**r = 0.182** ***p* = 0.044**
**Waist circumference**	**r = −0.477** ***p* < 0.001**	Creatinine	r = 0.045 *p* = 0.623
**Hip circumference**	**r = −0.441** ***p* < 0.001**	Cystatin C	**r = 0.288** ***p* = 0.002**
**FFM**	**r = −0.335** ***p* < 0.001**	Urate	**r = −0.199** ***p* = 0.039**
**TBW**	**r = −0.316** ***p* = 0.001**	Total cholesterol	r = −0.013 *p* = 0.889
**ECW**	**r = −0.284** ***p* = 0.003**	LDL	r = −0.060 *p* = 0.515
**BCM**	**r = −0.350** ***p* < 0.001**	HDL	**r = 0.203** ***p* = 0.027**
**FM**	**r = −0.484** ***p* < 0.001**	Triglycerides	**r = −0.220** ***p* = 0.016**
**PA**	**r = −0.307** ***p* = 0.001**	Vitamin D	r = 0.116 *p* = 0.210
**Systolic pressure**	**r = −0.442** ***p* < 0.001**	Homocysteine	r = −0.086 *p* = 0.367
**Diastolic pressure**	r = −0.142 *p* = 0.117	Urinary albumin/creatinine	**r = 0.318** ***p* = 0.001**
**Pulse wave velocity**	r = −0.047 *p* = 0.661		
**Liver elastography**	**r = −0.184** ***p* = 0.041**		
**Left kidney elastography**	**r = −0.355** ***p* < 0.001**		
**Right kidney elastography**	**r = −0.292** ***p* = 0.001**		

**Table 3:** Multivariate regression with IL-2Rα as independent factor.

Dependent Variable	Beta Coefficient	Significance
Systolic pressure	−2.748	0.011
Diastolic pressure	+0.050	0.973
Creatinine	+0.468	0.488
Cystatin C	+204.676	0.021
Urinary albumin/creatinine	+0.077	0.894
Body mass index	−4.946	0.040

## Data Availability

All the data are available from the corresponding author upon request. The data are not publicly available due to privacy considerations.
